# Characterizing Aciniform Silk Repetitive Domain Backbone Dynamics and Hydrodynamic Modularity

**DOI:** 10.3390/ijms17081305

**Published:** 2016-08-10

**Authors:** Marie-Laurence Tremblay, Lingling Xu, Muzaddid Sarker, Xiang-Qin Liu, Jan K. Rainey

**Affiliations:** 1 Department of Biochemistry & Molecular Biology, Dalhousie University, Halifax, NS B3H 4R2, Canada; mltremblay@dal.ca (M.-L.T.); ln217415@dal.ca (L.X.); muzaddid.sarker@dal.ca (M.S.); paul.liu@dal.ca (X.-Q.L.); 2 Department of Chemistry, Dalhousie University, Halifax, NS B3H 4R2, Canada

**Keywords:** aciniform spidroin (AcSp1), wrapping silk, recombinant spider silk, modular proteins, reduced spectral density mapping, hydrodynamics characterization, nuclear magnetic resonance spectroscopy, split intein, segmental-labelling

## Abstract

Spider aciniform (wrapping) silk is a remarkable fibrillar biomaterial with outstanding mechanical properties. It is a modular protein consisting, in *Argiope trifasciata*, of a core repetitive domain of 200 amino acid units (W units). In solution, the W units comprise a globular folded core, with five α-helices, and disordered tails that are linked to form a ~63-residue intrinsically disordered linker in concatemers. Herein, we present nuclear magnetic resonance (NMR) spectroscopy-based ^15^N spin relaxation analysis, allowing characterization of backbone dynamics as a function of residue on the ps–ns timescale in the context of the single W unit (W_1_) and the two unit concatemer (W_2_). Unambiguous mapping of backbone dynamics throughout W_2_ was made possible by segmental NMR active isotope-enrichment through split intein-mediated *trans*-splicing. Spectral density mapping for W_1_ and W_2_ reveals a striking disparity in dynamics between the folded core and the disordered linker and tail regions. These data are also consistent with rotational diffusion behaviour where each globular domain tumbles almost independently of its neighbour. At a localized level, helix 5 exhibits elevated high frequency dynamics relative to the proximal helix 4, supporting a model of fibrillogenesis where this helix unfolds as part of the transition to a mixed α-helix/β-sheet fibre.

## 1. Introduction

Spider aciniform (or wrapping) silk is the toughest type of silk and is a remarkable biomaterial with outstanding mechanical properties [1]. Spider silk proteins (spidroins) and silkworm silk proteins (fibroins) share a general architecture of a relatively long repetitive domain, comprising a concatenated series of repetitive units or sequence motifs, flanked by much shorter non-repetitive N- and C-terminal domains [2,3]. Aciniform spidroin (AcSp1) is the primary constituent of wrapping silk. In *Argiope* *trifasciata*, it is a modular protein containing at least 14 identical concatenated repeats of a 200 amino acid unit (termed here “W” units, from wrapping) [4].

Modular protein architecture, in which discrete structured modules are connected together by linkers that range from rigid to highly flexible, is common in nature [5,6,7]. The structure of individual domains are frequently studied in isolation by nuclear magnetic resonance (NMR) spectroscopy and/or X-ray diffraction, then placed in a multi-domain context through NMR spectroscopy [8,9], small angle X-ray scattering [10], or cryo-electron microscopy [11], allowing delineation of structure and dynamics in the context of the larger assembly. The orientation of domains relative to one another, their dynamics, and the relation between domains is crucial for expanding our understanding of their function [9,12,13].

Many multi-domain proteins comprise discrete, differing units (e.g., scaffolding units such as the SH2, SH3, PDZ, or PTB domains) connected by linkers with both widespread pathophysiological consequences [14] and potential for recombination for synthetic biology purposes [15]. From an NMR spectroscopy standpoint, di-ubiquitin [16,17] and GB1 [18] have been extensively studied as model multi-domain proteins. In contrast to these proteins, where discrete modules impart individual function, many fibrous proteins including spider silks employ repetitive modules [2]. This adds unique difficulties for structural biology, where the repetitive nature of these proteins leads to challenges in unambiguously tracking individual modules.

We recently employed NMR spectroscopy to determine the solution-state structure of the recombinant W unit of *A. trifasciata* AcSp1 in the context of both the single unit (W_1_) and the two-unit concatemer (W_2_) [19]. W_1_ is composed of a predominantly helical globular domain composed of five defined α-helices with an unstructured ~12 residue N-terminal tail and ~50 residue C-terminal tail. In W_2_, the tails of neighbouring units become a linker that retains intrinsically disordered behaviour while the globular domains are identically structured giving rise to beads-on-a-string type conformation.

Fibres cannot be formed from solutions of W_1_, but manual drawing of fibres is readily possible from solutions containing W_2_, W_3_, or W_4_ concatemers [20], including from solution-state NMR samples of W_2_ [19]. During fibre formation, AcSp1 undergoes a partial conversion from α-helical to β-sheet structuring [21], putatively seeded at helix 5 in the W unit [19,22]. This transition is recapitulated in recombinant W_2_ between the soluble and fibrous forms [19]. The 200 residue W unit from *A. trifasciata* differs significantly from other spider silks, such as the extensively studied major and minor ampullate silks, where short repetitive motifs such as A*_n_*, (GA)*_n_*, GGX or GPGXX dominate the protein sequence [1,2]. Silkworm fibroin is also dominated by short motifs (e.g., GAGAGS and GAGAGY) in its repetitive domain [3,23]. Echoing these differences in primary structuring, the retention of α-helical character in aciniform silk fibre is distinct from both ampullate silks and silkworm silk, where fibres are completely depleted of α-helical character [1,2,21]. Hence, although recombinant W proteins are much shorter than native aciniform silk proteins and lack non-repetitive N- and C-terminal domains, with reduced strength and extensibility relative to native silk that scale approximately with the number of W units [24], the structural behaviour of these proteins is consistent with the native protein.

Characterization of dynamics within proteins at the atomic-level is possible at a variety of time-scales using NMR spectroscopy [25,26]. Measurement of ^15^N nuclear spin relaxation properties, namely the longitudinal and transverse relaxation times (*T*_1_ and *T*_2_, respectively) and cross-relaxation through the heteronuclear ^1^H-^15^N nuclear Overhauser effect ([^1^H]-^15^N NOE) in particular, allow for characterization of small-amplitude, high-frequency motions as a function of position along the polypeptide backbone together with delineation of regions experiencing slower dynamic fluctuations [27]. Relation of these spin relaxation parameters to global and local motion is often carried out through the model-free [28] or extended model-free [29] approaches. In instances where a single global correlation time is not suitable, such as for proteins containing large unstructured regions, the reduced spectral density mapping approach provides residue-by-residue characterization of dynamics without a reliance on global rotational diffusion parameters [30].

These dynamics characterization methods rely upon unambiguous distinction of ^1^H-^15^N cross-peaks in 2D spectra, a situation impossible in concatemeric AcSp1 repeat units without some means to distinguish one W unit from the other. The technique of intein-mediated *trans*-splicing provides such a means, where individual W_1_ units can be selectively labelled with NMR active isotopes and investigated, in the present work, in the context of the larger fibre-forming W_2_ protein. Inteins are naturally-occurring protein segments that excise themselves from a polypeptide and ligate the flanking protein segments together with a native peptide bond. This reaction will occur provided that a nucleophilic Ser or Cys is present immediately C-terminal to the intein and that the intein-protein fragment pair are stable in solution and allow the ligation to occur [31,32].

There have now been many demonstrated applications of inteins in structural biology and biotechnology [32,33], including protein cyclization [34,35,36]; protein switches [37,38,39,40]; in vivo protein engineering and probe attachment for biophysical studies [41,42,43]; and, importantly for the present work, segmental isotope enrichment [19,31,44,45,46,47,48]. In our previous structural studies [19], we performed segmental-labelling using split intein *trans*-splicing [49,50], whereby either the first (W_2-1_) or second (W_2-2_) W unit in W_2_ was enriched with NMR-active ^13^C and/or ^15^N nuclei while the other W unit was at natural abundance. Although we demonstrated differential dynamics between the globular core and linker/tail regions through variation in the observed heteronuclear [^1^H]-^15^N NOE, a more in-depth analysis of backbone dynamics is necessary to compare and contrast the behaviour of isolated W units vs. concatemers and to provide insight into more subtle variations in dynamics within the W unit.

Herein, new insight has been gained into the modularity, global conformation, and localized backbone dynamics behaviour of spider wrapping silk concatemers through characterization of ps–ns timescale NMR relaxation behaviour of each W unit in W_2_ relative to one another and to W_1_. In W_1_ and in each W unit of W_2_, reduced spectral density mapping is consistent with a structured five α-helix globular core having elevated dynamics in helix 5 with intrinsically disordered N- and C-terminal tails and, in W_2_, linker. Nuclear spin relaxation data are consistent with rotational diffusion by a compact globular core in W_1_ and with modular tumbling of each W unit in W_2_ almost independently of the remainder of the protein. Beyond ramifications for AcSp1 behaviour, the methods presented herein will serve as a useful atomic-level model for further characterization of modular proteins in solution.

## 2. Results

### 2.1. Nuclear Spin Relaxation Parameters

**Figure 1:**
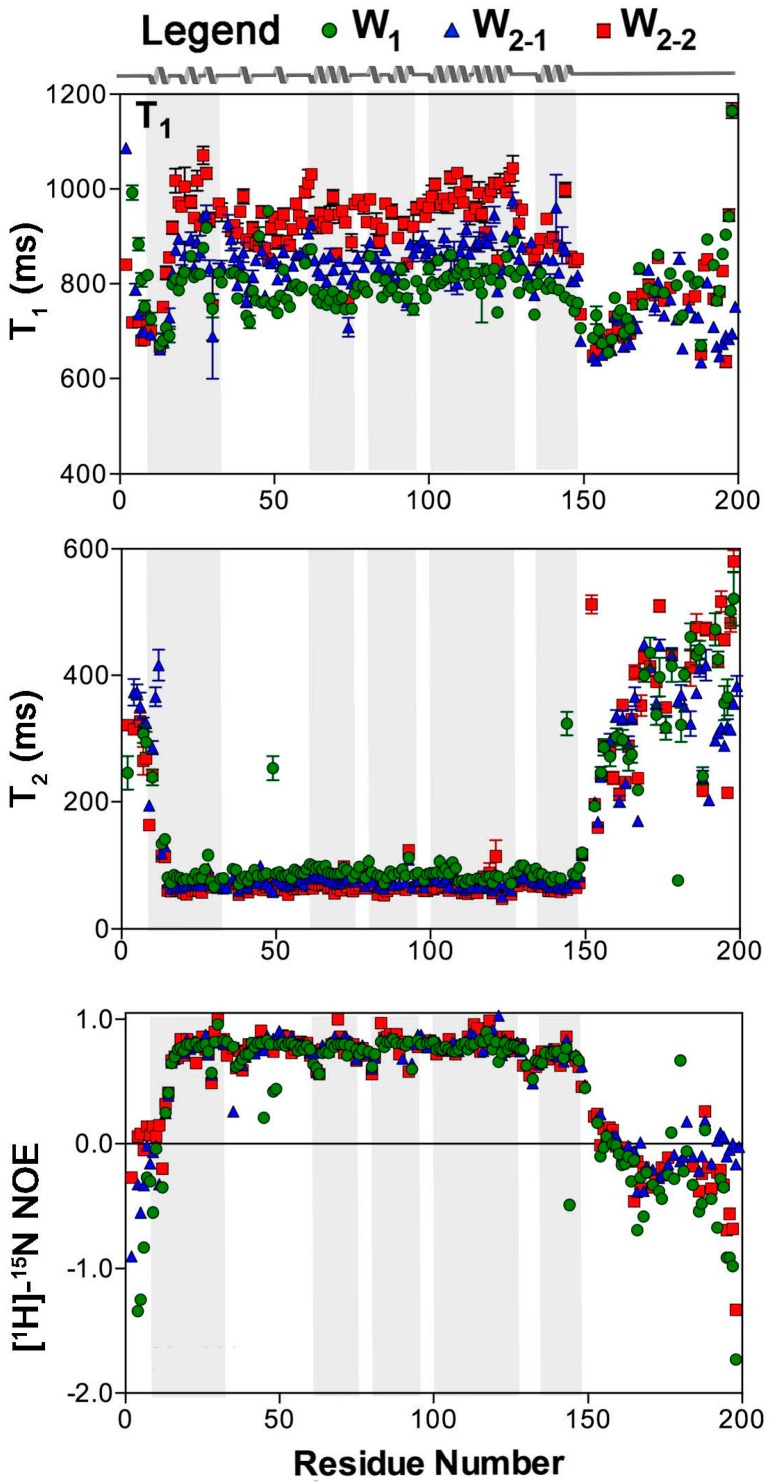
^15^N spin relaxation parameters as a function of residue at 16.4 T for W_1_, W_2-1_, and W_2-2_. Analysis and error propagation were carried out using Mathematica notebooks from Leo Spyracopoulos [51]. The secondary structuring of the W unit is depicted on the basis of PDB entry 2MU3 [19], with grey shading for each helical segment.

Longitudinal (*T*_1_) and transverse (*T*_2_) relaxation time constants were measured at 16.4 T on a residue-by-residue basis [27] for both the monomeric (W_1_) and concatemeric (W_2_) states of recombinant AcSp1 (Figure 1). To facilitate direct comparison, and because these data are integral for the subsequent analysis, the [^1^H]-^15^N data that we previously reported [19] are also plotted in Figure 1. A segregation in spin relaxation behaviour is clear between (i) the folded domain (residues 12–149 for a given W unit, with secondary structure elements shown in linear form in Figure 1) and (ii) the N- and C-terminal tails of W_1_ and W_2_ and the linker spanning W_2-1_ to W_2-2_ in W_2_. Namely, *T*_1_ and the [^1^H]-^15^N NOE are elevated through the folded core of a given W unit and decrease in the linker and tail regions while *T*_2_ exhibits the opposite trend.

**Figure 2:**
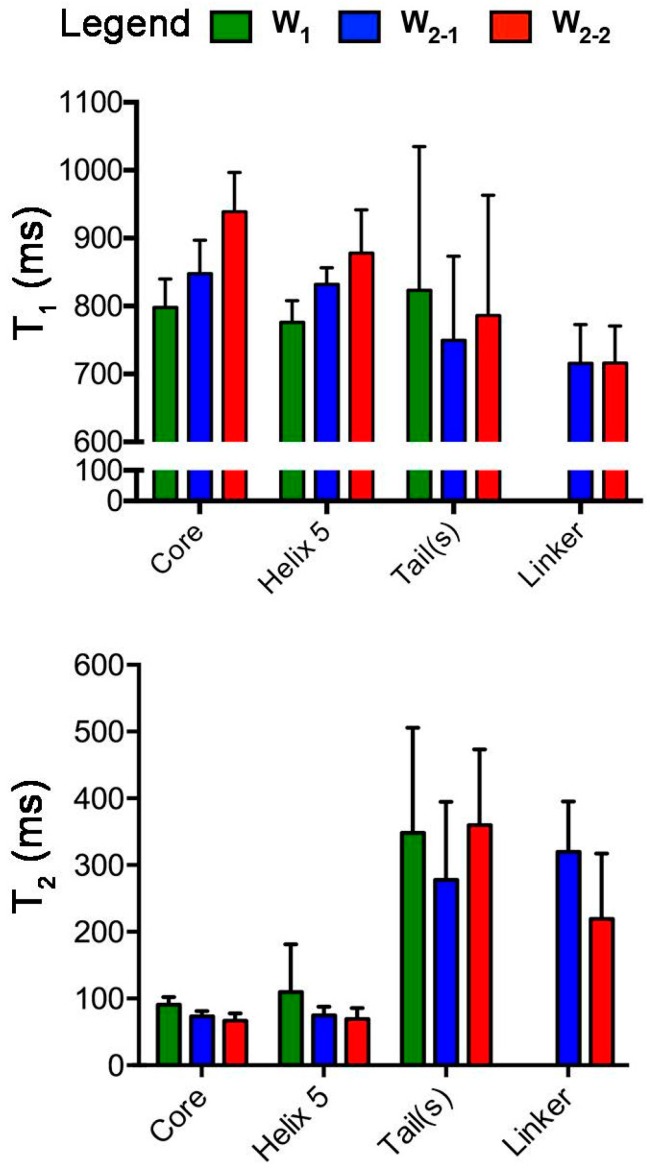
Bar graphs representing the mean *T*_1_ and *T*_2_ (error bars: standard deviations) for key regions of the W unit. These were specifically defined as the core (residues 12–149 (W_1_, W_2-1_) and 212–349 (W_2-2_)), linker (residues 150–211 from W_2-1_ and W_2-2_), tails (W_1_: residues 1–11 and 150–199; W_2_: residues 1–11 and 350–400), and helix 5 (residues 135–149 (W_1_, W_2-1_) and 335–349 (W_2-2_)).

For direct overall comparison, mean values of *T*_1_ and *T*_2_ were determined for four subdivided regions of the W unit chosen based upon our previous structural and ^19^F-NMR studies: the globular core (residues 12–149 and, in W_2_, 212–349), helix 5 within the core (residues 135–149 and, in W_2_, 335–349), tails (W_1_: residues 1–11 and 150–199; W_2_: residues 1–11 and 350–400), and the linker (residues 150–211 in W_2_ only) (Figure 2). Qualitatively, *T*_1_ is larger while *T*_2_ is smaller in the globular core for each W_2_ subunit relative to W_1_. The observed behaviour is consistent with the ^15^N relaxation behaviour to be expected on the basis of more rapidly (W_1_) vs. slowly (W_2_) tumbling molecules [27]. Notably, *T*_1_ values for W_1_, W_2-1_, and W_2-2_ are significantly different for the core (*p*-value < 0.0001) and helix 5 (*p*-value < 0.01), while the tails are relatively similar (albeit with large variance) regardless of protein size.

In examining overall *T*_2_ behaviour (Figure 2), the most striking feature is the large difference in mean values between the globular core and the disordered tails/linker, with a significant elevation in *T*_2_ for the tails or linker in all W units. As with *T*_1_, W_1_ exhibits a significant difference in behaviour from both W_2-1_ and W_2-2_ (*p*-value < 0.0001), with an elevated *T*_2_ relative to either unit in W_2_. Although the significance is low (*p*-value < 0.1), helix 5 follows the same qualitative trend. Unlike with *T*_1_, however, W_2-1_ and W_2-2_ do not exhibit significant differences in *T*_2_ relative to each other. Although the tails would be expected to be less encumbered and more dynamic overall, there is no difference between the mean values observed for the tails and the linker.

### 2.2. Reduced Spectral Density Mapping

**Figure 3:**
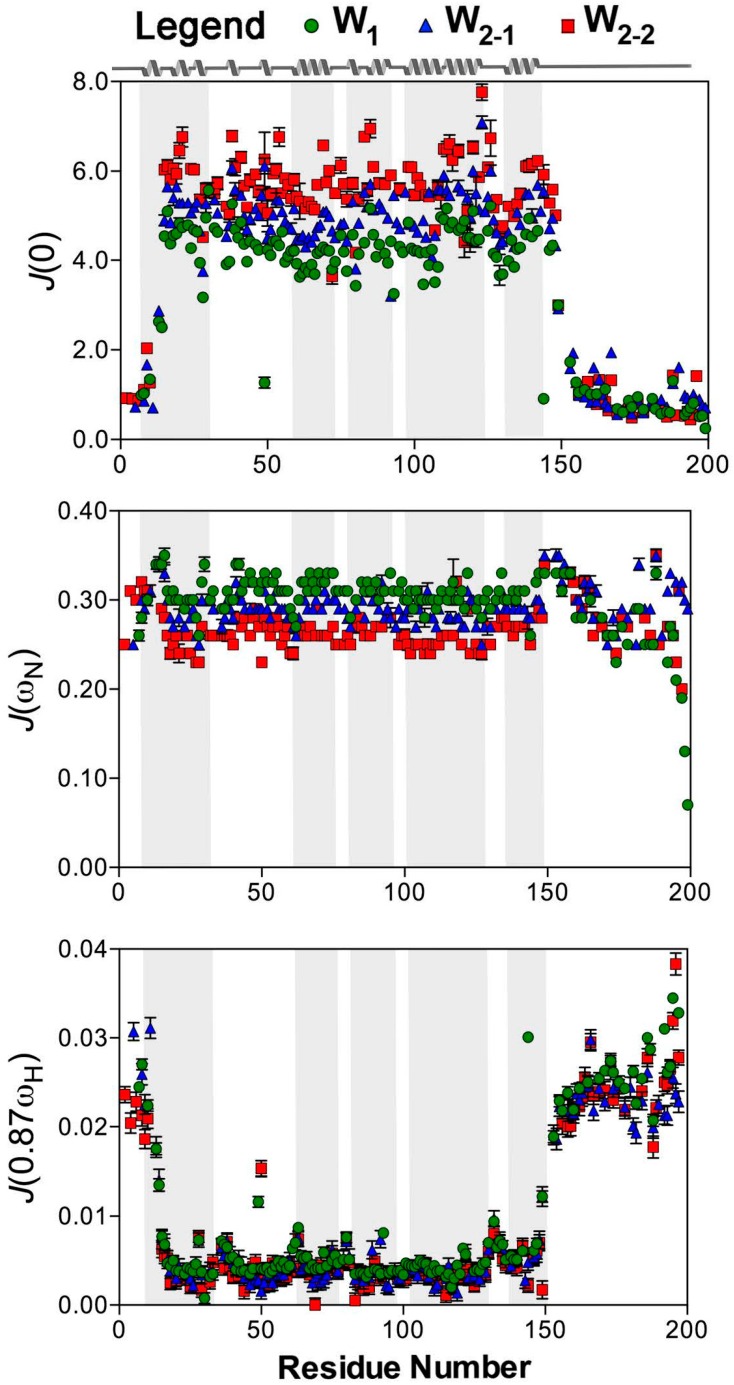
^15^N reduced spectral density mapping [30] at 16.4 T as a function of residue for W_1_, W_2-1_, and W_2-2_. Analysis and error propagation were carried out using Mathematica notebooks from Leo Spyracopoulos [51].

**Figure 4:**
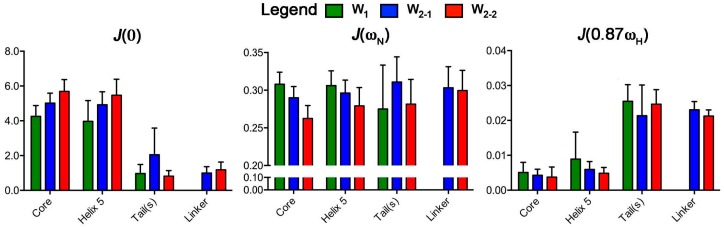
Bar graphs representing the mean *J*(0), *J*(ω_N_), and *J*(0.87ω_H_) (error bars: standard deviation) over key W unit regions. These were specifically defined as the core (residues 12–149 (W_1_, W_2-1_) and 212–349 (W_2-2_)), linker (residues 150–211 from W_2-1_ and W_2-2_), tails (W_1_: residues 1–11 and 150–199; W_2_: residues 1–11 and 350–400), and helix 5 (residues 135–149 (W_1_, W_2-1_) and 335–349 (W_2-2_)).

The values of the reduced spectral density at *J*(0), *J*(ω_N_), and *J*(0.87ω_H_) were calculated independently for each W unit. All residues with *T*_1_ and *T*_2_ fits that met the goodness-of-fit criterion (χ^2^ < critical χ^2^ [51]) for a given dataset at 16.4 T were employed for spectral density determination, giving 145, 153, and 131 residues, respectively, for W_1_, W_2-1_, and W_2-2_. *J*(0.87ω_H_) and *J*(0) both strongly demonstrate disparate dynamics between the folded core and the tails/linker, mirroring the behaviour of the individual *T*_1_, *T*_2_, and [^1^H]-^15^N NOE parameters (Figure 3 and Figure 4). *J*(ω_N_), conversely, remains relatively uniform throughout all regions of a given unit of W_1_ or W_2_, without significant differences between globular and linker domains. Reflecting the relatively strong dependence of *J*(ω_N_) on *T*_1_, an expected decrease in *J*(ω_N_) is observed from W_1_, W_2-1_, to W_2-2_ for the globular core (*p*-value < 0.0001), with helix 5 following suit (*p*-value < 0.01), while those for the tails and linker are not significantly different between the W units.

*J*(0.87ω_H_), which is very sensitive to differences in motion in the high frequency regime [52], exhibits localized increases in W_1_ and in each W unit of W_2_ around residues 36, 63, 80, 121, and 132 (Figure 3), correlating directly to the locations of loops or turns within the W unit [19]. It should be noted, though, that the increases in *J*(0.87ω_H_) observed at these locations are not of the same magnitude as those seen for the linker or tails (Figure 3). These variations are, therefore, likely reflective of regions of the protein experiencing increased dynamics but tumbling with the core of the folded domain rather than behaving as intrinsically-disordered segments.

A statistically significant increase in *J*(0.87ω_H_) is also observed for helix 5 (residues 135–149) relative to helix 4 (residues 101–127) (*p*-values of 0.0060, 0.0001, and 0.0025 for W_1_, W_2-1_, and W_2-2_, respectively) or to the core (helices 1–4) (*p*-values of 0.019, 0.0011, and 0.0085 for W_1_, W_2-1_, and W_2-2_, respectively). Helix 1, like helix 5, is directly connected to a disordered tail or linker segment [19]; therefore, helix 1 would be expected to exhibit similarly elevated dynamics if proximity to a tail or linker were the only factor at play. Instead, helix 1 behaves more like a core helix, as demonstrated by a lack of significant differences in *J*(0.87ω_H_) for helix 1 vs. helix 4 (*p*-values of 0.11, 0.046, and 0.126 for W_1_, W_2-1_, and W_2-2_, respectively). There is also a qualitative difference between *J*(0.87ω_H_) of helix 5 in the W units, with W_1_ > W_2-1_ > W_2-2_ (Figure 4) following the same qualitative trend as the core as a whole.

Like *J*(ω_N_), the behaviour at *J*(0) differs between W_1_, W_2-1_, and W_2-2_ in the globular core (*p*-value < 0.0001) and helix 5 (*p*-value < 0.001), increasing from W_1_, to W_2-1_ and W_2-2_ and opposite in trend to the observed decrease in *J*(ω_N_). W_1_ has the lowest mean frequency at *J*(0) over the folded core (4.26 ± 0.07), followed by W_2-1_ (5.02 ± 0.11), and then W_2-2_ (5.67 ± 0.37) (Figure 4). On average, the tail/linker regions do differ between W_1_, W_2-1_, and W_2-2_ but care must be taken during interpretation given that the tail group for W_2-1_ has seven values and W_2-2_ linker has six values. When the tails and linker are grouped together, there is no statistical difference between the W units.

### 2.3. Analysis of Rotational Diffusion

Spin relaxation data were used to compare the suitability of isotropic, axially symmetric, or fully anisotropic rotational diffusion tensors for W_1_, W_2-1_, W_2-2_, and W_2_ based upon all residues with [^1^H]-^15^N NOE > 0.65 [53] using the software ROTDIF [54]. In each instance, an axially symmetric rotational diffusion tensor provided the best fit to the data (Table 1); notably, the degree of anisotropy observed was minimal in light of the fact that ~90% of an 878 monomeric protein dataset exhibited an anisotropy of 1.17 or higher [54]. W_1_ spin relaxation data were best fit with a prolate rotational diffusion tensor for all 20 members of the NMR structural ensemble (PDB entry 2MU3 [19]), with an anisotropy range of 1.09–1.18. Conversely, W_2-2_ was uniformly oblate (anisotropy 0.90–0.95 with W_1_ ensemble) while W_2-1_ varied depending upon the ensemble member employed, with anisotropy of 1.05–1.16 (16 members) or 0.88–0.95 (four members) observed. The fitting behaviour for W_2-1_ is consistent with the small degree of anisotropy observed, with minimal deviation from an isotropic fit. Additionally, diffusion tensors were modelled for a combined W_2-1_ and W_2-2_ spin relaxation data set using the ensemble of 20 inferred W_2_ structures [19]. In this instance, anisotropy remained minimal and most ensemble members led to oblate fits (anisotropy 0.8–0.92 for 18 members; 1.12–1.13 for two members). Goodness-of-fit, as judged by χ^2^ per degrees of freedom, was comparable in all instances (Table 1).

Ensemble-averaged values of τ_c_ based upon anisotropic diffusion tensors demonstrated only modest increases from 7.9 ns for W_1_ to 9.0 ns for W_2-1_ to 9.6 ns for W_2-2_. To place this behaviour in context, a variety of rotational correlation time (τ_c_) estimations were compared (Table 2). Using Stokes’ law (Equation (5)), crude values of τ_c_ were estimated both according to an assumption of spherical shape (Equation (6)) and according to our previously reported hydrodynamic radii determined by diffusion ordered NMR spectroscopy (DOSY) and verified by dynamic light scattering [19]. Our NMR-derived W_1_ structural ensemble and the inferred W_2_ structural ensemble were also used for detailed hydrodynamics calculations in HYDROPRO [55] to estimate τ_c_. To test the effect of a more compact globular tumbling unit, τ_c_ values were also determined for the W_1_ globular core (i.e., excluding the N- and C-terminal tails) with and without the inclusion of the more dynamic (Figure 4) and less stable helix 5 [19,22]. It should also be noted that the viscosities employed for the Stokes’ law and HYDROPRO hydrodynamics calculations (Table 2) were experimentally determined, rather than estimated. This determination was carried out through DOSY experiments with use of an internal dioxane standard [56]. Estimated τ_c_ values for W_1_ were most consistent with the experimentally-observed behaviour for the most compact estimates of its conformation. W_2_, conversely, was estimated regardless of the hydrodynamic model employed to tumble much more slowly than was experimentally observed for each globular unit in the concatemer.

The ratio of *T*_1_ to *T*_2_ can also be related to τ_c_ for the tumbling of a macromolecule in solution under the qualification that the ^1^H-^15^N spin pair in question does not experience significant rapid internal motion [27]. Extending this treatment, measured backbone relaxation time constants may be modified (giving *T*_1_’ and *T*_2_’, Equations (1) and (2), respectively) to remove high frequency spectral density contributions [17]. The ratio of these modified relaxation time constants, ρ (Equation (3)), is relatively insensitive to localized variations in dipolar coupling and ^15^N chemical shift anisotropy and, for a protein core, primarily dependent on overall tumbling. Direct comparison of the estimated τ_c_ obtained from average values of *T*_1_/*T*_2_ or *T*_1_′/*T*_2_′ on the basis of the ^15^N relaxation analysis of Kay et al. [27] neglecting high-frequency terms (Equation (7)) demonstrates excellent agreement with the far more rigorous ROTDIF calculation.

**Figure 5:**
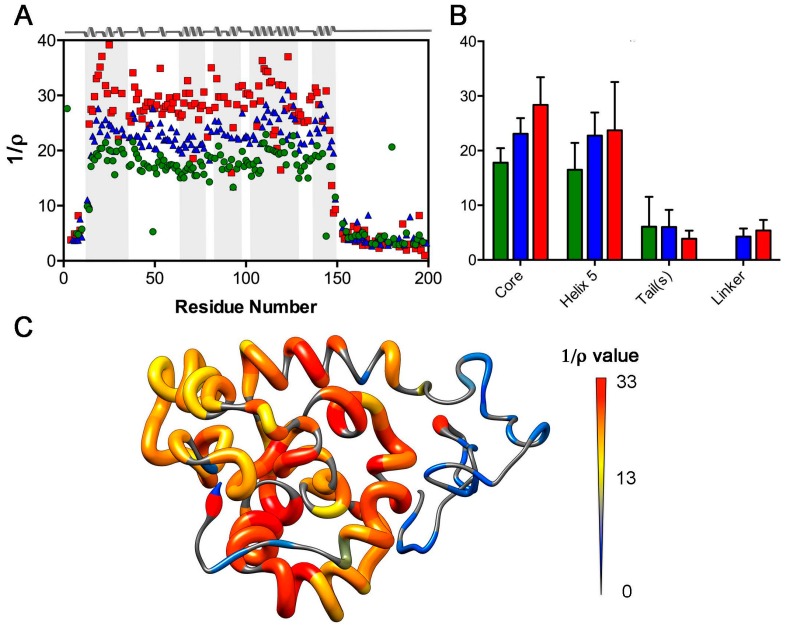
Inverse of ρ ratio of *T*_1_’ to *T*_2_’ (relaxation times modified to remove high-frequency components of spectral density) as defined by Fushman et al. [17]. (**A**) Plotted as a function of residue (green circles: W_1_; blue triangles: W_2-1_, red squares: W_2-2_) with secondary structuring of the W unit depicted on the basis of PDB entry 2MU3 [19] using grey shading to delineate each helical segment (**B**) bar graphs for mean (green: W_1_; blue: W_2-1_, red: W_2-2_; error bars: standard deviation) over key W unit regions. These were specifically defined as the core (residues 12–149 (W_1_, W_2-1_) and 212–349 (W_2-2_)), linker (residues 150–211 from W_2-1_ and W_2-2_), tails (W_1_: residues 1–11 and 150–199; W_2_: residues 1–11 and 350–400), and helix 5 (residues 135–149 (W_1_, W_2-1_) and 335–349 (W_2-2_)); and (**C**) plotted for W_1_ on the lowest-energy NMR ensemble member of PDB entry 2MU3 [19], with both backbone thickness and colour varied as a function of 1/ρ, as indicated.

At a more global level, discrete differences in 1/ρ are apparent between the globular core vs. tail and linker regions of W_1_, W_2-1_, and W_2-2_ (Figure 5), with the core of W_1_ being significantly decreased in 1/ρ (*p-*value < 0.0001) relative to W_2-1_ or W_2-2_ and mirroring of this behaviour by helix 5 (*p*-value < 0.01) (Figure 5B). Following the same trend as *J*(0) (Figure 4), W_1_ has a statistically significantly lower mean (*p-*value < 0.0001) 1/ρ in the core compared to W_2-1_; W_2-1_ is again significantly lower than W_2-2_ (*p-*value < 0.0001), reflecting differences in rotational diffusion between W_1_ and each of the W units in W_2_ (Figure 5; Table 1). Additionally, regardless of being in a tail or linker, the corresponding residues in a given W unit in W_2_ (i.e., residues 1–11 relative to 201–211 and 150–200 relative to 350–400) demonstrate very similar mean 1/ρ values. The variance accompanying 1/ρ is, however, too great to draw significance.

## 3. Discussion

AcSp1 from *A. trifasciata* is a modular protein composed primarily of a repetitive domain of concatenated 200 amino acid W units [4]. We recently demonstrated that the W unit is composed of a well-folded globular domain of ~138 residues connected to adjacent globular domains by intrinsically-disordered linkers ~62 residues in length [19]. The functional necessity of this folded domain is further implied from the fact that it appears to be highly conserved (albeit considering a limited number of sequenced species), while the linker may vary both in length and sequence [57].

The modularity of AcSp1 was established through direct backbone chemical shift comparison between the monomeric (W_1_) and concatemeric (W_2_) states of AcSp1. Specifically, the chemical shifts of W_2_ are remarkably similar to W_1_ with exception of those in the linker immediately proximal to the covalent W unit linkage [19]. Beyond conservation of chemical shifts, heteronuclear [^1^H]-^15^N NOE data recorded at 16.4 T (Figure 1) also uphold the conformational independence of W units, given that W_1_ and each of the W units in W_2_ exhibit very similar NOE enhancement factor patterns as a function of position within the W unit [19]. In each case, higher NOE enhancement factors are exhibited in the folded domain (residues 12–149, numbering relative to each W unit) and lower or negative enhancements in the disordered terminal or linker regions (residues 1–11, 150–200) (Figure 1). The effect of concatemeric linking of W units is observed in the vicinity of the covalent linkage of the W units (residues ~190 to 210 of W_2_) through a less negative NOE enhancement relative to the free N- and C-terminal tails of W_1_ and of W_2-1_ and W_2-2_, respectively.

Our previous studies showed clear modularity in the W unit both in terms of structuring of the globular domain and the intrinsic disorder of the linker. The ^15^N spin relaxation measurements and reduced spectral density mapping detailed herein demonstrate that this modularity clearly extends beyond structuring and into the dynamic behaviour along the polypeptide backbone. Segmental isotope-labelling mediated by split intein *trans*-splicing allowed us to track this behaviour unambiguously along the length of W_2_. It should be noted that the relaxation analysis methods employed herein are limited to probing motions in the ps-ns regime [26], but that the *trans*-splicing methodology, itself, can be directly applied to other NMR-based methods allowing characterization of longer time-scale motions.

Direct comparison of the N- and C-terminal W units in the concatemer was, thus, possible alongside a comparison of W_1_ to W_2_. *T*_1_, *T*_2_, [^1^H]-^15^N NOE, and 1/ρ, in each case, explicitly delineate the globular vs. tail or linker domains (Figure 1, Figure 2 and Figure 5). For the most part, the globular domain exhibits uniform spin relaxation behaviour, with slight localized decreases in the [^1^H]-^15^N NOE delineating the secondary structure elements centred at residue 35–37 (between helix 1 and the converged, predominantly helical region over residues 40–60), residue 61 (between residues 40–60 and helix 2), residues 79–80 (between helices 2 and 3), residues 91–93 (near the helix 3 C-terminus), and residues 128–132 (between helices 4 and 5).

Given the existence of discrete disordered and globular domains in the W unit, we employed the ^15^N reduced spectral density mapping approach [30] to analyze backbone dynamics, rather than the model-free [28] or extended model-free [29] formalism. This approach alleviates the requirement for defining specific motional modes and their independence or lack thereof, with demonstrated suitability for proteins of mixed ordered and disordered segments [52]. The resulting values of the spectral density function at three frequencies, *J*(0), *J*(ω_N_), and *J*(0.87ω_H_), derived from relaxation parameters *T*_1_, *T*_2_, and [^1^H]-^15^N NOE provide complimentary information along the peptide backbone (Figure 3 and Figure 4) and unequivocally support our original model of W_2_, based on W_1_ restraints [19], with the tail/linker regions and the globular domain being noticeably segregated.

*J*(ω_N_) is much less sensitive to internal motions at the ps-ns time scale in comparison to *J*(0) and *J*(0.87ω_H_) and displays very little variability between the folded domain and the linker. The residues in the folded domain have large *J*(0) and small *J*(0.87ω_H_), while the linker and tail regions display the opposite trend (Figure 3 and Figure 4). This is consistent with a situation where the linker and tails experience motion over a wider range of frequencies relative to the globular domain, as would be expected for an intrinsically disordered domain. Noticeably, the mean *J*(0) and *J*(ω_N_) values over the globular domain differ significantly between W_1_, W_2-1_, and W_2-2_, increasing or decreasing, respectively, from W_1_ to W_2-1_ to W_2-2_. This behaviour is consistent with an increase in tumbling rates from W_1_ to W_2-1_ to W_2-2_.

Based upon both heteronuclear NMR [19] and ^19^F-NMR [22], helix 5 (residues 135–149) and the portion of the globular domain in contact with it (falling in proximity to residue 36) are less stable than the remainder of the protein. Chaotropic denaturation or treatment with the detergent dodecylphopshocholine lead to helix 5 destabilization and a concomitant structural rearrangement in the globular core of the W unit [19,22]. Notably, therefore, the spectral density in helix 5 deviates from the remainder of the globular domain. Direct comparison to the proximal helix 4 shows elevated spectral density at *J*(0.87ω_H_). *J(*0) is also qualitatively lower for helix 5 than for the remainder of the globular core (helix 1–4) (Figure 3). This behaviour, as a whole, is consistent with a greater sampling of high-frequency motion in this region of the W unit regardless of whether it is in an isolated W unit or a concatemeric construct.

Helix 5 falls immediately N-terminal to the intrinsically disordered linker. Our working hypothesis is that decompaction of the W unit occurs through loss of interaction of helix 5 with the core [22] followed by denaturation [19]. This would, in turn, greatly favour protein-protein entangling and interaction, inducing subsequent β-sheet formation during fibrillogenesis. Backbone-level dynamics are consistent with the distinct behaviour of helix 5 relative to the remainder of the globular domain and unambiguously demonstrate a propensity for increased high frequency motion.

Before considering rotational tumbling behaviour in more detail, it should be noted that the viscosities measured for the W_1_ and W_2_ samples in NMR buffer (Table 2) are significantly higher than the ~0.82 cP that is derived on the basis of a linear combination of the expected [58] H_2_O and D_2_O viscosities at 30 °C. The source of this elevated viscosity is not fully clear. Given that W_1_ will, for example, spontaneously form nanoparticle (or micellar) structures in aqueous solution [59], supramolecular assembly was certainly a distinct possibility. Neither spin relaxation behaviour (Table 2) nor translational diffusion observed by DOSY [19] are consistent with long-lived entanglement of the proteins or of stable oligomer formation. Were entanglement, oligomerization, or nanoparticle/micelle formation happening in the bulk of the sample, substantially slower tumbling and diffusion than observed would be expected. The fact that the vast majority of protein in solution is still fully observable on the basis of both spin relaxation behaviour and signal intensity by heteronuclear ([19] and herein) and ^19^F [22] NMR implies that if intermolecular entangling and/or supramolecular assembly are occurring and increasing solution viscosity that this only involves a small fraction of the total protein.

Tumbling of W_1_, reflected in the observed τ_c_ of 7.9 ns, is more rapid than would be anticipated strictly on the basis of the W_1_ hydrodynamic radius previously determined though DOSY [19] or through hydrodynamics calculations using the W_1_ structural ensemble (Table 2). W_1_, instead, exhibits tumbling consistent with a compact spherical particle of the same molecular weight with a half-shell of water. The overestimates in τ_c_ on the basis of overall W_1_ shape and dimensions are not surprising, given that the presence of intrinsically disordered domains in a protein leads to a general overestimation of τ_c_ by methods (such as HYDROPRO) that employ an assumption of rigid behaviour [60,61]. Truncation of the W_1_ structure either to the globular core or to the core without helix 5 lead to improved agreement between the inferred and observed τ_c_ values, with the helix 1–4 globular core leading to a predicted τ_c_ of ~8.4 ns. Rotational diffusion is, therefore, most consistent with a compact globular core where helix 5 is not always attached. Translational diffusion, conversely, agrees well with the overall shape of W_1_ [19].

Modest increases in τ_c_, to 9.0 ns for W_2-1_ and 9.6 ns for W_2-2,_ are observed relative to 7.9 ns for W_1_. These values are ~2/3 of those predicted for a compact sphere and ~1/2 those predicted on the basis of the DOSY-determined W_2_ hydrodynamic radius and <1/3 that predicted by HYDROPRO on the basis of the W_2_ structural ensemble. This behaviour is also directly reflected in the magnitude of the observed increases in 1/ρ from W_1_ to W_2-1_ to W_2-2_ (Figure 5). Namely, W_2_ does not exhibit anywhere near the expected [17] ~doubling of 1/ρ relative to W_1_ that would be observed if the two W units in W_2_ were rigidly tumbling together as a species of double the molecular weight. Following studies using ensemble methods to accurately predict rotational diffusion for molecules containing intrinsically-disordered linkers [61,62], this is instead consistent with mostly decoupled tumbling of each globular domain. The increased τ_c_ of W_2-2_ relative to W_2-1_ is consistent with greater hydrodynamic friction experienced from the asymmetric nature of the tails, with an ~11 residue disordered N-terminal tail for W_2-1_ vs. an ~50 residue disordered C-terminal tail for W_2-2_.

## 4. Materials and Methods

### 4.1. Sample Preparation

Protein samples were prepared by recombinantly expressing W_1_ and W_2_ in *Escherichia coli* BL21(DE3), following previously-described protocols [19,63]. It should be noted that W_1_ consists of residues 1–199 of the AcSp1 repeat unit from *A. trifasciata* while W_2-1_ and W_2-2_ each comprise the full 200 amino acid repeat unit concatenated to form a 400 residue protein. An N-terminal Met is also present in W_2_ from the initiation codon; for simplicity of comparison between W_1_ and each unit in W_2_, the Met is not included in residue numbering. Uniformly ^15^N-enriched W_1_ (~0.2 mM), and selectively ^15^N-enriched W_2-1_ and W_2-2_ (~0.2 mM) NMR samples were prepared in sodium acetate buffer (20 mM *d*_3_-acetate (Sigma-Aldrich Canada, Oakville, ON, Canada) in H_2_O:D_2_O (Sigma-Aldrich Canada) at 90%:10% (*v*/*v*), 1 mM NaN_3_ (Sigma-Aldrich Canada), 1 mM 2,2-dimethyl-2-silapentane-5-sulfonic acid (DSS) (Wilmad, Buena, NJ, USA); pH 5).

### 4.2. Spin-Relaxation NMR Experiments

NMR spin relaxation experiments were carried out at 30 °C on an Avance III NMR spectrometer operating at 16.4 T (Bruker Canada, Milton, ON, Canada) and equipped with a triple-resonance 5 mm indirect detect TCI cryoprobe. Two-dimensional phase-sensitive ^1^H-^15^N HSQC experiments were used to measure longitudinal relaxation times (*T*_1_; hsqct1etf3gpsi pulse program, Bruker library) and transverse relaxation times (*T*_2_; hsqct2etf3gpsi pulse program, Bruker library). All experiments were performed using 16 scans, 1.5 s recycle delay for W_1_ and 1.75 s for W_2-1_ and W_2-2_, spectral widths of 23 and 16 ppm with offsets of 115.5 ppm and at the water frequency (4.705 ppm), respectively, for ^15^N and ^1^H. W_1_ spectra contained 192 × 2048 complex points and W_2-1_ and W_2-2_ contained 128 × 2048 complex points for the ^15^N and ^1^H, respectively. The *T*_1_ data were collected using relaxation delays of 50, 100, 250, 500, 750, 1000, 1300, and 1700 ms and the *T*_2_ data were collected using 17, 34, 51, 85, 119, 152, 187, and 238 ms relaxation delays, with a Carr-Purcell-Meiboom-Gill pulse train applied as appropriate for a given relaxation delay during the recycle delay to compensate for heating effects. [^1^H]-^15^N steady-state heteronuclear nuclear Overhauser effects ([^1^H]-^15^N NOE; hsqcnoef3gpsi pulse program, Bruker library) for the ^15^N nuclei were measured in an interleaved manner as described previously [19]. Briefly, the [^1^H]-^15^N NOE measurements were performed using a total of 356 × 4096 complex points with 32 transients for W_1_ and 256 × 4096 complex points and 32 transients for both W_2_ domains.

### 4.3. Determination of Spin Relaxation Parameters and Reduced Spectral Density mapping

Backbone ^15^N *T*_1_, *T*_2_, and [^1^H]-^15^N NOE as a function of ^1^H-^15^N cross-peak position were determined and correlated to our previously assigned chemical shifts (deposited in the Biological Magnetic Resonance Data Bank for W_1_ (BMRB entry 17899) and W_2_ (BMRB entry 25197) [19,63]). The ^15^N *T*_1_ and *T*_2_ values with associated errors were determined using the Mathematica version 8.0.4 (Wolfram, Champaign, IL, USA) notebook *Relaxation Decay*, freely available from Leo Spyracopoulos [51]. *R*_1_ (*R*_1_ (s^−1^) = 1/*T*_1_) and *R*_2_ (*R*_2_ (s^−1^) = 1/*T*_2_) relaxation rates were determined from nonlinear least-square fits to a two-parameter monoexponential decay. Errors were estimated based on the average spectral noise. The [^1^H]-^15^N heteronuclear NOE was measured as the ratio of the saturated spectrum to the reference spectrum as *I*_sat_/*I*_ref_ where *I*_sat_ and *I*_ref_ are the intensities of the peaks in the ^1^H-^15^N HSQC spectra, with and without proton saturation during the recycle delay, respectively. Non-linear fits were used to minimize the statistical value of χ^2^. The χ^2^ goodness-of-fit test per residue was used and compared to the exact critical χ^2^ determined from 100 Monte Carlo simulations (9.146) for a single residue at a 95% confidence interval:

1/*T*_1_’ = *R*_1_’ = *R*_1_[1 − 1.249|γN/γH|(1 − NOE)] (1)

and:

1/*T*_2_’ = *R*_2_’ = *R*_2_ − 1.079|γ_N_/γ_H_|*R*_1_(1 − *NOE*) (2)

where γ_N_ and γ_H_ are the gyromagnetic ratios of ^15^N and ^1^H, respectively. The ratio of these modified rates, was calculated as:

ρ = [(2*R*_2_’/*R*_1_’) − 1]^−1^ (3)

Finally, per-residue values of *J*(0), *J*(ω_N_), and *J*(0.87ω_H_) were determined through ^15^N reduced spectral density mapping [30] using the *Spectral Density* Mathematica notebook [51].

### 4.4. Viscosity Determination

The viscosity (η) of each NMR sample was calculated using a dioxane internal standard [56]. DOSY experiments acquired and processed as detailed previously for W_1_ and W_2_ [19] were analyzed to directly determine the translational diffusion coefficient (*D*_C_) for dioxane in a given W sample. Coupling each measured D_C_ with the known hydrodynamic diameter (*d*_H_) of dioxane (0.424 nm [56]), η may be determined through the Stokes-Einstein equation [64]:

*D*_C_ = (k_B_T)/(3πη*d*_H_) (4)

where k_B_ is the Boltzmann constant and T the absolute temperature (303 K).

### 4.5. Analysis of Rotational Diffusion

To analyze rotational diffusion behaviour with respect the ^15^N spin relaxation data, isotropic, axially symmetric, and anisotropic diffusion tensors models were applied to W_1_, W_2-1_, and W_2-2_ using ROTDIF 3.1 [54]. Only residues with [^1^H]-^15^N NOE > 0.65 and not likely to be involved in conformational exchange were used for the analysis. For W_1_, W_2-1_, and W_2-2_, the 20-member W_1_ structural ensemble (PDB ID 2MU3) was iteratively analyzed through ROTDIF using robust least-square fitting to obtain global information with coordinates from the lowest energy member to model the diffusion tensor frame (D_||_ and D_⊥_ tensor axes for an axially symmetric system, or D_xx_, D_yy_, and D_zz_ tensor axes for a fully anisotropic system) and Euler angles (α, β, and/or γ). In addition, the W_2_ ensemble member with the calculated R_g_ closest to the experimental R_g_ was deemed the representative model for the reference frame of the diffusion tensor for W_2_ (merged W_2-1_ and W_2-2_ relaxation data). The robust least-squares optimization method was employed during fitting and full statistical analysis was employed to determine the most statistically upheld diffusion tensor model for a given ensemble member.

### 4.6. Estimations of Rotational Correlation Time

The rotational correlation time (τ_c_), assuming a hydrated sphere, may be estimated through Stokes’ law [64]:

τ_c_ = (4πη*r*_H_^3^)/(3k_B_T) (5)

where *r*_H_ is the radius of hydration. For a hydrated protein, *r*_H_ may be roughly estimated on the basis of the specific volume (υ = 0.73 cm^3^/g) as [65]:

*r*_H_ = [3υ*M*_r_/(4π*N*_A_)]^1/3^ + *r*_w_ (6)

where *M*_r_ is the molecular weight, *N*_A_ is Avogadro’s number, and *r*_w_ is radius of the hydration layer surrounding the protein (1.6–3.2 Å for ½-1 hydration shell [66]).

For direct comparison, the average ratios of *T*_1_/*T*_2_ (or *T*_1_′/*T*_2_′) for all residues with an NOE > 0.65 in a given protein were employed to estimate τ_c_. Through neglecting of the high-frequency terms of the spectral density, the analysis of Kay et al. [27] may be simplified to:

τ_c_ = [1/(4πν_N_)] (6*T*_1_/*T*_2_ – 7)^1/2^ (7)

where ν_N_ is the resonance frequency of ^15^N (in Hz).

HYDROPRO [55] was also used to estimate ensemble-averaged τ_c_ values based upon the NMR-derived structural ensemble for W_1_ (PDB entry 2MU3) and the W_2_ ensemble inferred on the basis of concatenated NMR-derived restraints for W_1_ [19]. The resulting output was parsed for τ_c_ (harmonic mean (correlation) time) as calculated on the basis of the combined input of temperature, solvent viscosity, molecular weight, solute partial specific volume, solution density, and PDB structural coordinates. For comparison, calculations were carried out for W_1_ structural ensembles truncated using an in-house Tcl/Tk script to the globular core (residues 12–149) or the globular core excluding both the turn between helices 4 and 5 and helix 5 (residues 12–128).

### 4.7. Statistical Tests

Statistical analyses were performed between units and protein regions as described above to evaluate significance between means through ordinary one-way ANOVA test when comparing 3 or more means or unpaired two tailed *t*-test with Welch’s correction for unequal variances when comparing two means within the Prism 6 or InStat software packages (both from GraphPad Software Inc., La Jolla, CA, USA). All distributions were assumed to be Gaussian. Unless otherwise noted, significance was determined at an α of 0.05.

## 5. Conclusions

The core repetitive domain of AcSp1 is composed of concatenated 200 amino acid units, identical in sequence and very similar in tertiary structuring and internal motions. Through split intein-mediated *trans*-splicing, individual repeat units were selectively isotope-enriched and investigated in the context of the W_2_ protein capable of fibrillogenesis. Intein-mediated segmental-labelling is also highly promising for future studies of other modular proteins, whereby spectral complexity can be reduced without compromising the functional state of the protein. Backbone-level dynamics very clearly demonstrate the beads-on-a-string conformation of the AcSp1 repetitive domain, with structured globular domains linked by lengthy intrinsically-disordered segments forming a relatively viscous solubilized state. Although our previous translational diffusion studies imply that the linker is not highly extended, with W_2_ and W_3_ exhibiting relatively compact conformations, the ^15^N spin relaxation behaviour detailed herein demonstrate that each globular domain in W_2_ tumbles nearly independently of its neighbour. Regardless of the construct examined, helix 5 also exhibited elevated high-frequency dynamics relative to the remainder of the globular core. Rotational diffusion behaviour of W_1_ is also most consistent with a W unit globular core where helix 5 is not stably attached. Unambiguous measurement of backbone dynamics, therefore, improves our understanding of both AcSp1 repetitive domain modularity and allow direct demonstration of variations in localized stability that were implied by titration with chaotropes and detergent.

## Figures and Tables

**Table 1:** Rotational diffusion tensor parameters that best fit indicated ^15^N spin relaxation data set.

Protein ^1^	N–H Bonds ^2^	D_⊥_ (×10^−7^ s^−1^)	D_||_ (×10^−7^ s^−1^)	Anisotropy	α (°)	β (°)	τ_c_ (ns)	χ^2^/df ^3^
W_1_	102	2.01 ± 0.05	2.30 ± 0.08	1.14 ± 0.17	13 ± 52	43 ± 19	7.91 ± 0.03	1.409
W_2-1_	104	1.82 ± 0.07	1.93 ± 0.04	1.06 ± 0.13	164 ± 87	138 ± 20	8.98 ± 0.03	1.889
W_2-2_	93	1.78 ± 0.03	1.63 ± 0.03	0.92 ± 0.09	77 ± 37	155 ± 10	9.65 ± 0.04	1.906
W_2_	197	1.87 ± 0.04	1.68 ± 0.04	0.90 ± 0.11	26 ± 31	20 ± 22	9.24 ± 0.05	2.309

^1^ Diffusion tensor detailed for lowest-energy member of the W_1_ structural ensemble (PDB entry 2MU3 [19]) for W_1_, W_2-1_, and W_2-2_; and, for member of inferred [19] W_2_ structural ensemble with radius-of-gyration closest to that determined by diffusion ordered NMR spectroscopy for W_2_; ^2^ Residues having [^1^H]-^15^N NOE > 0.65 used in ROTDIF [54] fit; ^3^ df: degrees of freedom (equivalent to number of N–H bonds).

**Table 2:** Rotational correlation time (τ_c_) for indicated protein according to given method.

Protein	η (cP) ^1^	τ_c_ (ns)					
		Stokes (Ideal) ^2^	Stokes (DOSY) ^3^	Structure ^4^	*T*_1_/*T*_2_^5^	*T*_1_′/*T*_2_′ ^5^	ROTDIF ^6^
W_1_	1.040	7.8–9.9	10.6	14.1 ± 0.3 ^7^ 9.4 ± 0.2 ^8^ 8.4 ± 0.1 ^9^	7.9	8.0	7.9 ± 0.01
W_2-1_	1.056	-	-	-	9.0	9.1	9.0 ± 0.01
W_2-2_	1.056	-	-	-	9.5	9.6	9.6 ± 0.01
W_2_	1.056	14.7–17.8	19.7	31.8 ± 0.7	9.3	9.4	9.3 ± 0.03

**^1^** Viscosities determined using dioxane internal standard by DOSY (Equation (4)); ^2^ Calculated using Stokes’ law (Equation (5)) for a 100% ^15^N/^13^C-enriched protein mass using a hydration shell of either 1.6 Å (lower estimate) or 3.2 Å (upper estimate), assuming spherical shape (Equation (6)); ^3^ Calculated using Stokes’ law (Equation (5)) based upon hydrodynamic radii determined by DOSY [19]; ^4^ Average ± average deviation of HYDROPRO [55] predicted τ_c_ over 20-member ensembles of structures of W_1_ or W_2_ [19], or over globular core of W_1_; ^5^ Determined based upon indicated relaxation time constant ratio using Equation (7); ^6^ Average ± average deviation over all 20 structural ensemble members determined through axially-symmetric diffusion tensor ROTDIF in identical manner to Table 1; ^7^ Entire W_1_ structure; ^8^ Globular core (residues 12–149); and, ^9^ Globular core excluding helix 5 (residues 12–128).
